# Physiological and Biochemical Mechanisms of Wood Vinegar-Induced Stress Response against Tomato Fusarium Wilt Disease

**DOI:** 10.3390/plants13020157

**Published:** 2024-01-06

**Authors:** Hongyin Zhou, Kejian Fu, Yan Shen, Runhe Li, Youbo Su, Yishu Deng, Yunsheng Xia, Naiming Zhang

**Affiliations:** 1 College of Plant Protection, Yunnan Agricultural University, Kunming 650201, China; zhy1605202632@163.com (H.Z.); 18314457137@163.com (Y.S.); 2 College of Resources and Environment, Yunnan Agricultural University, Kunming 650201, China; fkj0524@126.com (K.F.); llrunhe@163.com (R.L.); youbosu@ynau.edu.cn (Y.S.); yshengxia@163.com (Y.X.); 3 College of Architectural Engineering, Yunnan Agricultural University, Kunming 650201, China; 13987627051@163.com

**Keywords:** wood vinegar, tomato, biotic stress, growth, disease resistance mechanism

## Abstract

Wood vinegar, a by-product of charcoal biomass pyrolysis, has been used as a biofungicide in plant disease management because of its antimicrobial properties. However, the physiological and biochemical mechanisms through which wood vinegar alleviates biotic stress are poorly understood. In this study, pot experiments were conducted to investigate the resistance and regulation mechanism of wood vinegar prepared from different raw materials (ZM) and from a single raw material (SM) in controlling tomato (*Solanum lycopersicum* “Bonny Best”) Fusarium wilt at different concentrations (0.3%, 0.6%, 0.9%, 1.2%, and 1.5%). The results showed that ZM and SM had significant control effects on tomato fusarium wilt under different concentrations in the same growth cycle. Under biotic stress, the two kinds of wood vinegar significantly increased the plant height, stem diameter, leaf area and yield of tomato under the concentration of 0.3%, 0.6%, 0.9% and 1.2%, and significantly reduced the content of malondialdehyde (MDA) and hydrogen peroxide (H_2_O_2_) in tomato leaves. The effect of 0.9% treatment was the most significant, ZM and SM significantly increased tomato yield by 122% and 74%, respectively, compared with CK under 0.9% treatment. However, the plant height, stem diameter and leaf area of tomato were significantly reduced under 1.5% treatment, but the content of soluble sugar, soluble protein and vitamin C in tomato fruit was the best. Compared with CK, ZM significantly increased by 14%, 193% and 67%, respectively, and SM significantly increased by 28%, 300% and 159%, respectively. Except for 0.3% treatment, both significantly increased the activities of catalase (CAT), peroxidase (POD) and superoxide dismutase (SOD) in tomato leaves. The response intensity of two kinds of wood vinegar—physiological and biochemical—to tomato disease resistance, growth and development, showed ZM > SM. The disease index of tomato showed highly significant negative correlation with plant height, stem thickness, leaf area and antioxidant physiology CAT, and highly significant positive correlation with MDA and H_2_O_2_ content. In conclusion, ZM was more effective than SM in enhancing tomato disease resistance by promoting tomato growth and development, decreasing leaf MDA and H_2_O_2_ content, and inducing antioxidant enzyme activity in leaves at moderate concentrations.

## 1. Introduction

Owing to climate change, energy shortages, environmental pollution, food crises, and limited sustainable development of agriculture, biochar-based agricultural and forestry wastes are widely used in agriculture for energy generation and environmental conservation because of their role in carbon sequestration, emission reduction, and increasing carbon sinks [1]. With the increasing demand for biochar in recent years, the amount of lignocellulosic biomass pyrolysis liquid produced during biochar preparation has also increased. Biomass pyrolysis liquids are known as wood vinegar. As a by-product, wood vinegar is a new pollutant. Therefore, there is an urgent need to investigate the functions of wood vinegar, which has become a popular research topic in recent years [2,3]. Wood vinegar has a special smoked aroma and sour taste, and is generally yellowish brown or dark brown, with a pH ranging between 1.5 and 3.7, and specific gravity greater than 1.005 [4,5]. Wood vinegar is a complex mixture with 80–90% water as the main component containing more than 200 water-soluble compounds, including nitrogen, phenols, organic acids, sugar derivatives, alcohols, and esters [6,7]. The chemical composition of wood vinegar is primarily determined by the heating rate, temperature, and raw materials used [8]. As a natural agricultural product, wood vinegar does not pollute the environment and has no toxic effects on humans or animals. Therefore, the properties of wood vinegar have gradually been recognized and valued, and it is widely used in agriculture, forestry, animal husbandry, and other fields [9,10]. Wood vinegar has been used extensively in agriculture, mainly for soil improvement, promotion of plant growth, such as vegetables and fruit trees, improvement of fruit taste and quality, and replacement of synthetic pesticides. As a new type of green natural product, wood vinegar not only degrades easily, but also contains acids and active phenolic substances with insecticidal and antibacterial effects [11].

Tomato (*Solanum lycopersicum*) is one of the most important and widely cultivated vegetable crops worldwide but it is threatened by wilt disease during growth [12]. Tomato Fusarium wilt (TFW) is a soil-borne fungal disease caused by *Fusarium oxysporum*. When tomatoes are infected with *F. oxysporum*, they exhibit symptoms, including reduced root elongation, poor development, leaf yellowing from bottom to top, water loss, and reduced fruit yield, among others, which causes substantial economic losses related to tomato production [13]. Infection by the fungus also manifests physiologically as an accumulation of reactive oxygen species (ROS) in cells [14]. When the generated reactive oxygen species exceed the ability of the corresponding scavenging system, they can inhibit chlorophyll synthesis in crops, cause membrane lipid peroxidation, and damage DNA, proteins, and other biological molecules [15]. *F. oxysporum* is transmitted through plants, seeds, soil, dead leaves, irrigation water, and agricultural facilities, and it has a wide range of pathogenic variants, making it difficult to control and prevent [16]. Currently, TFW is primarily controlled using synthetic pesticides. Although the use of these synthetic fungicides is effective and targeted and has achieved optimal control of plant diseases, excessive use of the fungicides can be harmful to human health, lead to loss of biodiversity, environmental pollution, and enhanced fungal resistance because of their high toxicity [17,18,19]. The side effects of these synthetic pesticides negatively impact the environment and niches [20,21,22]. Currently, sustainable agricultural guidelines prohibit the use of large amounts of synthetic pesticides for crop and fruit production [23,24]. Therefore, it is important to explore alternative methods that are safe, efficient, and environmentally friendly, in line with regulatory requirements.

Wood vinegar is largely used in plant disease management because of its antibacterial properties [25,26]. Different concentrations of wood vinegar considerably affect crop diseases [27,28]. However, most studies on the prevention and control of crop diseases using wood vinegar have been limited to wood vinegar obtained from a single raw material. Few studies have investigated the prevention and control of crop diseases using wood vinegar prepared from various raw materials, and the associated mechanisms remain unclear. Wood vinegar diluted less than 300 times exerts substantial preventive and therapeutic effects on potted tomato plants infected with TFW [29]. Therefore, this study aimed to investigate the effect of different concentrations of wood vinegar prepared from a variety of raw materials (ZM) and from a single raw material (SM) on TFW, and the physiological and biochemical mechanisms of wood vinegar in alleviating TFW using pot experiments. Specifically, we aimed to determine (1) whether ZM is more effective than SM in controlling TFW, and (2) whether ZM is more effective than SM in promoting tomato growth and development, improving the nutritional quality of tomato fruits, reducing the degree of membrane lipid peroxidation, and enhancing antioxidant enzyme activity in tomato leaves under biotic stress. Based on the objectives, we hypothesized that (1) ZM has a stronger effect on TFW than SM; and (2) ZM is more effective than SM in promoting tomato growth and development, enhancing the nutritional quality of tomato fruits, attenuating membrane lipid peroxidation, and increasing antioxidant enzyme activity in tomato leaves under biotic stress.

## 2. Results

### 2.1. Disease Severity

**Figure 1:**
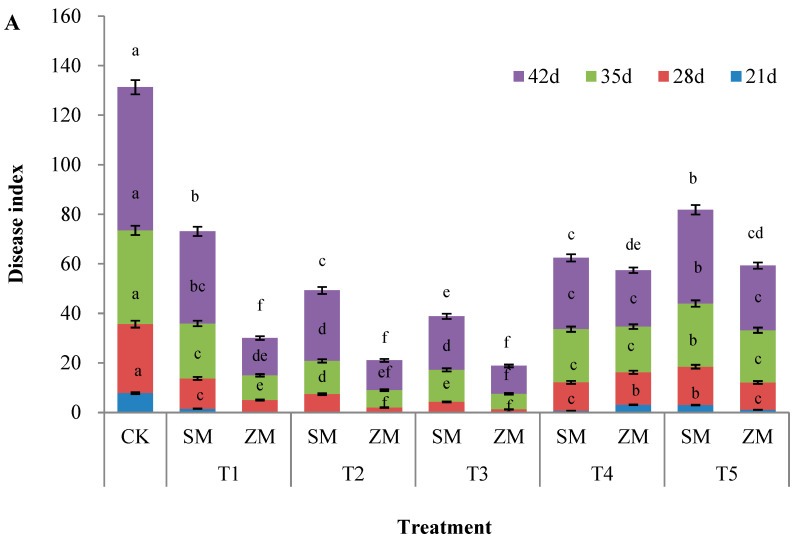

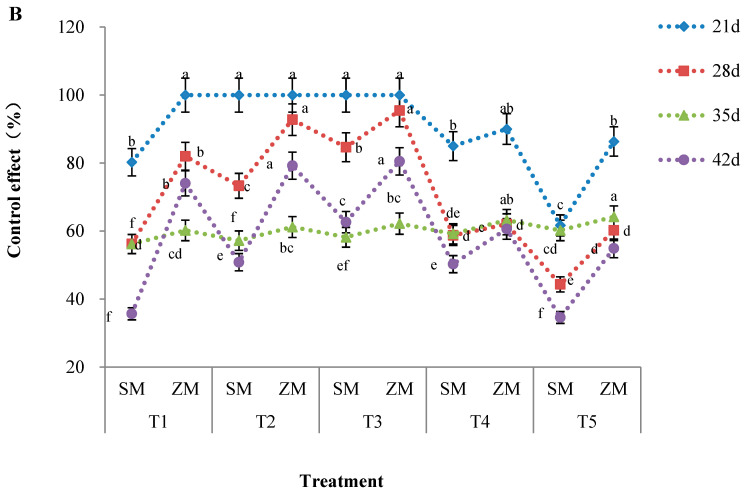
Control of tomato Fusarium wilt by two types of wood vinegar (SM and ZM). (**A**) Disease index and (**B**) control effect. SM: Wood vinegar prepared from a single raw material; ZM: Wood vinegar prepared from different raw materials. Data are the means of six replicates (means ± standard errors). Different lowercase letters indicate significant differences between the treatments at *p* < 0.05.

Disease severity of tomatoes under different treatments on the 21st, 28th, 35th, and 42nd days after inoculation with *F. oxysporum* is shown in Figure 1. The disease index of tomatoes treated with different concentrations of the two types of wood vinegar during the same growth cycle was significantly lower than that of the control. SM and ZM exerted significant effects on TFW under T3 treatment; compared with the control, T3 treatment of ZM significantly reduced disease index by 100%, 96%, 84% and 81% in the four growth cycles, respectively. Under the same concentration treatment, the disease index of ZM was significantly lower than that of SM. The results showed that the two types of wood vinegar had a certain controlling effect on TFW at different treatment concentrations. SM and ZM significantly enhanced disease resistance in tomato plants. SM and ZM had optimal effects on TFW under T3 treatment. The effect of ZM on TFW was stronger than that of SM, indicating that wood vinegar prepared using various raw materials has a stronger effect on enhancing disease resistance in tomatoes than wood vinegar prepared using a single raw material.

### 2.2. Plant Growth

As shown in Table 1, the two types of wood vinegar had different effects on tomato growth under biotic stress at different concentrations. After 21 d, 28 d, 35 d, and 42 d of tomato growth, SM and ZM significantly increased plant height, stem diameter, and leaf area of tomato plants in T1, T2, T3, and T4 treatments, respectively, when compared to CK, and the effect of the T3 treatment was the most significant; compared with the control, T3 treatment of ZM significantly increased the plant height by 67%, 80%, 62% and 43% in the four growth cycles of tomato, respectively, significantly increased stem diameter by 35%, 28%, 23% and 19%, and significantly increased leaf area by 122%, 105%, 91% and 74%. Furthermore, SM and ZM increased plant height, stem diameter, and leaf area of tomato plants in T1, T2, T3, and T4 treatments in the order of ZM > SM. However, a high concentration (1.5%) of SM and ZM significantly reduced tomato plant height and stem diameter in the T5 treatment when compared to that in CK, and the reduction effect was in the order of ZM > SM. This shows that certain concentrations of SM and ZM exhibit a good promoting effect on tomato growth and development under biotic stress, but high concentrations exert an inhibitory effect on tomato growth and development. The effect of wood vinegar prepared from various raw materials on tomato growth and development was stronger than that of wood vinegar prepared from a single raw material.

### 2.3. Nutritional Quality and Yield

The effects of different concentrations of the two types of wood vinegar on the nutritional quality of tomato fruits under biotic stress varied (Table 2). The different concentrations of the two types of wood vinegar significantly increased the soluble sugar, soluble protein, and vitamin C contents of tomato fruits in all treatments when compared to those in the CK, and the effect increased with an increase in treatment concentration. Significant increases were observed in T5 treatment. SM significantly increased soluble sugar, soluble protein, and vitamin C contents in T5 treatment by 14.23%, 192.74%, and 69.66%, respectively, when compared to those in CK. ZM significantly increased soluble sugar, soluble protein, and vitamin C contents in T5 treatment by 27.57%, 300%, and 159.11%, respectively, when compared to those in CK (Table 2). In addition, the enhancement effects of the two types of wood vinegar on soluble sugar, soluble protein, and vitamin C contents of tomato fruits were in the order of ZM > SM. The organic acid content of tomato fruits reduced significantly in all treatments, except for ZM, which significantly increased organic acid content in the T1 treatment when compared to that of CK (Table 2).

The effects of different concentrations of the two types of wood vinegar on tomato yield under biotic stress also differed (Table 2). In all treatments, compared with the control, ZM and SM had the largest increase in tomato yield under T3 treatment. Compared with CK, they were significantly increased by 122% and 74%, respectively. The results showed that both ZM and SM had the best effect on tomato yield under T3 treatment.

### 2.4. Malondialdehyde (MDA) Content in Tomato Leaves

**Figure 2:**
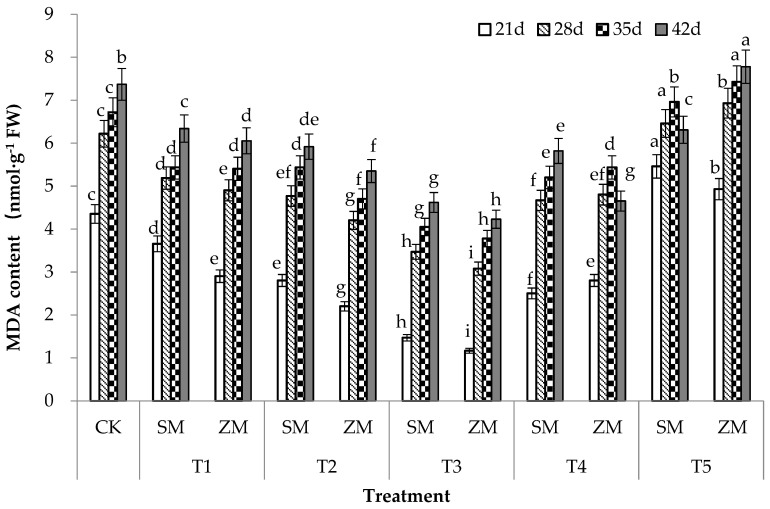
Effects of two types of wood vinegar (SM and ZM) on MDA content of tomato leaves. SM: Wood vinegar prepared from a single raw material; ZM: Wood vinegar prepared from different raw materials; MDA: Malondialdehyde. Data are the means of six replicates (means ± standard errors). Different lowercase letters indicate significant differences between the treatments at *p* < 0.05.

The different concentrations of the two types of wood vinegar had varied effects on the MDA content of tomato leaves in the same growth cycle under biotic stress (Figure 2). The effects of SM and ZM on the MDA content of tomato leaves first decreased and then increased with increasing treatment concentrations. SM and ZM significantly reduced the MDA content of tomato leaves in the T1, T2, T3, and T4 treatments when compared to that in CK, with the highest effect being observed in the T3 treatment; compared with the control, T3 treatment of ZM significantly decreased by 73%, 50%, 44% and 43% at 21 d, 28 d, 35 d and 42 d, respectively. The reduction effect on MDA content of tomato leaves in T1, T2, and T3 treatments was in the order of ZM > SM. High concentrations of SM and ZM significantly increased the MDA content of tomato leaves in the T5 treatment when compared to that in CK, and the increase followed the order of ZM > SM. The results suggest that the MDA content of tomato leaves increased sharply after tomatoes were infected with TFW, and the application of the two types of wood vinegar at a certain concentration alleviated membrane lipid peroxidation in tomato leaves. However, membrane lipid peroxidation in tomato leaves was aggravated at high treatment concentrations, with the effect of ZM on membrane lipid peroxidation being stronger than that of SM.

### 2.5. Hydrogen Peroxide (H_2_O_2_) Content in Tomato Leaves

**Figure 3:**
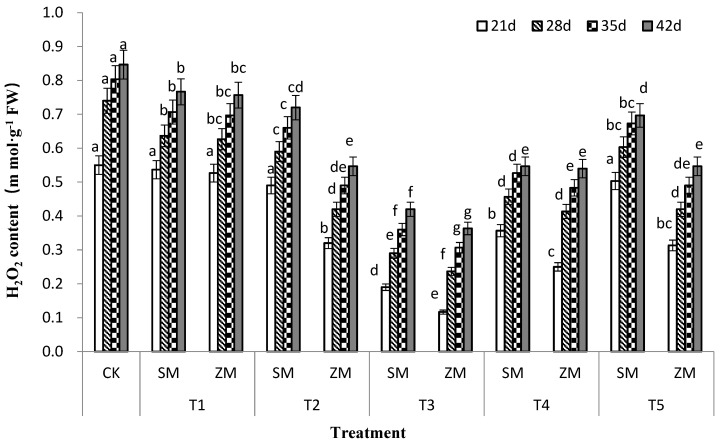
Effects of two types of wood vinegar (SM and ZM) on H_2_O_2_ content of tomato leaves. SM: Wood vinegar prepared from a single raw material; ZM: Wood vinegar prepared from different raw materials. Data are the means of six replicates (means ± standard errors). Different lowercase letters indicate significant differences between the treatments at *p* < 0.05.

The different concentrations of the two types of wood vinegar exerted varied effects on the H_2_O_2_ content of tomato leaves under biotic stress (Figure 3). SM and ZM significantly reduced the H_2_O_2_ content of tomato leaves when compared to CK, and the effect initially decreased and then increased with an increase in treatment concentration; the highest effect of SM and ZM was observed in T3 treatment. Furthermore, the reduction effect of SM and ZM on the H_2_O_2_ content of tomato leaves at the same treatment concentration was in the order of ZM > SM. Therefore, the application of the two types of wood vinegar at different concentrations can reduce the H_2_O_2_ content of tomato leaves, with the effect of ZM being stronger than that of SM.

### 2.6. Activities of Antioxidant Enzymes in Tomato Leaves

The antioxidant enzymes induced by biotic stress mainly comprise CAT, POD, and SOD. The results show that effects of the two types of wood vinegar on CAT, POD, and SOD activities in tomato leaves at different concentrations differed. The effects of SM and ZM on CAT activity in tomato leaves during the same growth cycle in T2, T3, T4, and T5 treatments initially exhibited an increasing trend and then decreased with increasing treatment concentrations. CAT activity in tomato leaves was the highest in T3 treatment; compared with the control, T3 treatment of SM significantly increased the activity by 28%, 14%, 17% and 16% at 21 d, 28 d, 35 d and 42 d, respectively. T3 treatment of ZM significantly increased the activity by 83%, 49%, 43% and 39% at 21 d, 28 d, 35 d and 42 d, respectively. Different concentrations of SM and ZM significantly increased POD and SOD activities in tomato leaves during the same growth cycle when compared to CK. POD and SOD activities in tomato leaves increased with an increase in treatment concentrations, and the highest POD and SOD activities in tomato leaves were observed in T5 treatment; compared with the control, T5 treatment of SM significantly increased POD activity by 274%, 161%, 121% and 119% in four growth cycles, respectively, and significantly increased SOD activity by 373%, 181%, 136% and 119%. T5 treatment of ZM significantly increased POD activity by 351%, 191%, 163% and 156% in four growth cycles, respectively, and significantly increased SOD activity by 397%, 193%, 145% and 126%.

### 2.7. Correlation Analysis

Analysis of correlations among disease index, agronomic traits (plant height, stem thickness, and leaf area), MDA content, H_2_O_2_ content, and antioxidant enzyme activity in tomatoes grown for 42 d (Table 3) showed that the disease index of tomatoes treated with the two types of wood vinegar was significantly negatively correlated with plant height, stem thickness, leaf area, and CAT activity, but significantly positively correlated with MDA and H_2_O_2_ contents. Analysis of correlations between ZM and SM concentrations with tomato disease index, agronomic traits (plant height, stem diameter, and leaf area), MDA content, H_2_O_2_ content, and antioxidant enzyme activity (Table 4 and Table 5) showed that ZM concentration was significantly positively correlated with tomato disease index and disease resistance-related enzyme activities (CAT, POD, and SOD activities), but significantly negatively correlated with plant height. A significant positive correlation was observed between SM concentration and disease resistance-related enzyme activities (POD and SOD activities). Therefore, the two types of wood vinegar improved disease resistance in tomatoes by promoting their growth and development, reducing oxidative damage to tomato leaves, and inducing antioxidant enzyme activity in tomato leaves. ZM exerted a more significant effect on tomato disease index, growth and development, and antioxidant enzyme activity in tomato leaves than SM.

## 3. Discussion

The results of this study showed that the disease index of tomatoes treated with different concentrations of the two types of wood vinegar was significantly lower than that of the control. The two types of wood vinegar had a beneficial effect on TFW, and they significantly enhanced disease resistance in tomato plants (Figure 1), which is consistent with the findings of previous studies [29]. Wood vinegar diluted less than 300 times has been shown to have a marked therapeutic effect on potted tomato plants infected with TFW. The application of appropriate concentrations of the two types of wood vinegar significantly promoted tomato growth and development under biotic stress (Table 1). The dual effects of wood vinegar on plant growth and the induction of secondary metabolites have been previously investigated. For example, Zhu et al. [30] suggested that an optimal concentration of wood vinegar can promote crop growth and enhance plant defense responses to stress conditions through the biosynthesis of active compounds. Wood vinegar not only enhances the accumulation of phenolic compounds with antioxidant activity in plants [31], but also considerably enhances the metabolism of terpenoids. Terpenoids are some of the main active molecules that enhance stress responses in plants [32]. In this study, tomato seedling growth was inhibited at a high concentration (1.5%) of the two types of wood vinegar (Table 1); however, the nutritional quality of tomato fruit improved substantially (Table 2). The observation is consistent with the results of Ofoe et al. [9], who found that spraying low concentrations (0.25% and 0.5%) of wood vinegar enhanced the morphological and physiological responses of the tomato plants. Spraying 2% wood vinegar can have a negative effect on tomato plants, although the concentration can significantly increase phytochemical content, such as total phenols and flavonoids, in tomato fruits, which improves their nutritional and health benefits. Moreover, the results of this study showed that the effect of wood vinegar prepared from different raw materials on TFW, and the morphological and physiological responses of tomato plants were stronger than those of wood vinegar prepared from a single raw material, and that high concentrations of wood vinegar had a contrasting effect. This could be associated with the different components of wood vinegar and the corresponding active ingredient content after pyrolysis of the different raw materials [33]. In this study, gas chromatography mass spectrometry (GC-MS) was used to analyze the chemical compositions of ZM and SM, and the results showed that their main components differed, as did their relative contents (Tables S1 and S2). Baharom et al. [34] studied antibacterial activities of wood vinegar derived from carambola, coconutshells, and mango against plant pathogenic microorganisms. All wood vinegars exhibit excellent antibacterial effects against pathogens and different wood vinegars exhibit varied antibacterial activities against pathogenic microorganisms [35]. Zhou et al. [36] showed that the effects of different wood vinegars on crop growth varied.

In addition, when plants are subjected to biotic stresses, they produce reactive oxygen species (ROS). The accumulated ROS in plants can directly attack unsaturated fatty acids in the membrane system, thereby initiating membrane lipid peroxidation [37]. MDA is the primary product of lipid peroxidation and its content can reflect the degree of damage to plants under stress [38]. The MDA content of plants increases after infection with pathogenic fungi [39], which is consistent with the results of this study. The MDA content of the tomato leaves under biotic stress in the control treatment was the highest. This content is related to the disease resistance of plants. A high MDA content indicates a high degree of infection by the pathogen and weaker resistance by plants [40]. This study showed that the two types of wood vinegar significantly reduced (*p* < 0.05) the disease index and MDA content of tomato leaves, which is consistent with the results of Chen et al. [41]. The incidence and degree of disease in grapes treated with 400-fold diluted wood vinegar were significantly reduced, and the lipid peroxidation rate in grape fruits was the lowest, with an optimal effect being observed. H_2_O_2_ is one of the ROSes produced in plants under biotic stress and it has two roles. As a signalling molecule involved in complex signal network transmission, H_2_O_2_ induces a series of defense mechanisms to protect plant cells from oxidative stress [42]. However, if excess H_2_O_2_ is not removed in time, it generates active hydroxyl radicals (•OH) through a metal-catalyzed Haber–Weiss reaction [43], resulting in plant damage. In this study, different concentrations of the two types of wood vinegars significantly reduced H_2_O_2_ content in tomato leaves (Figure 3).

**Figure 4:**
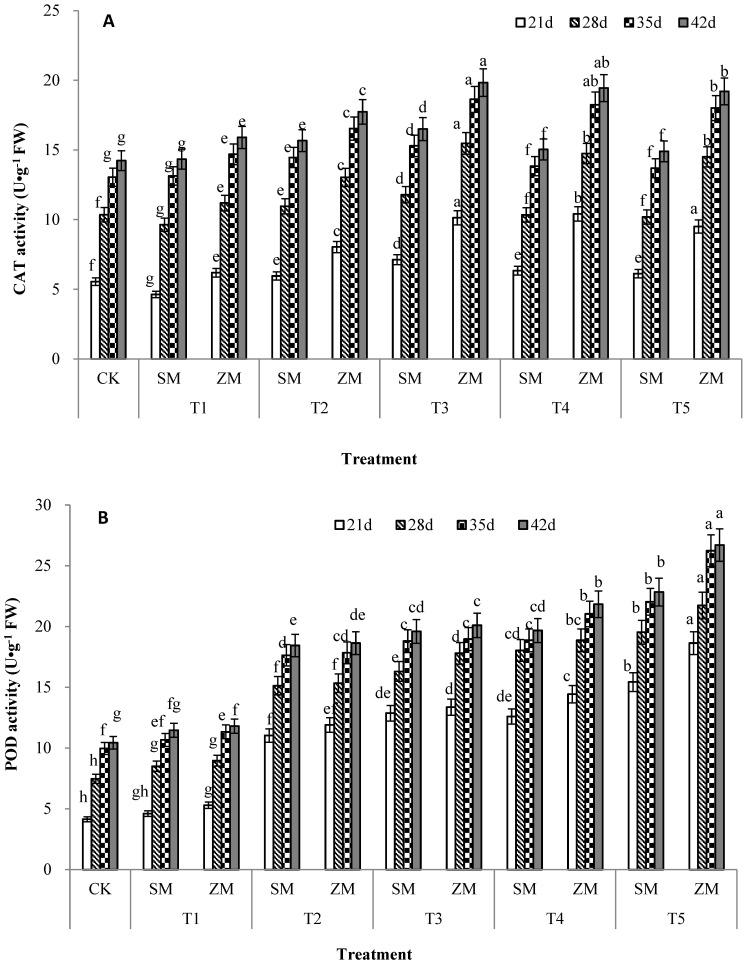

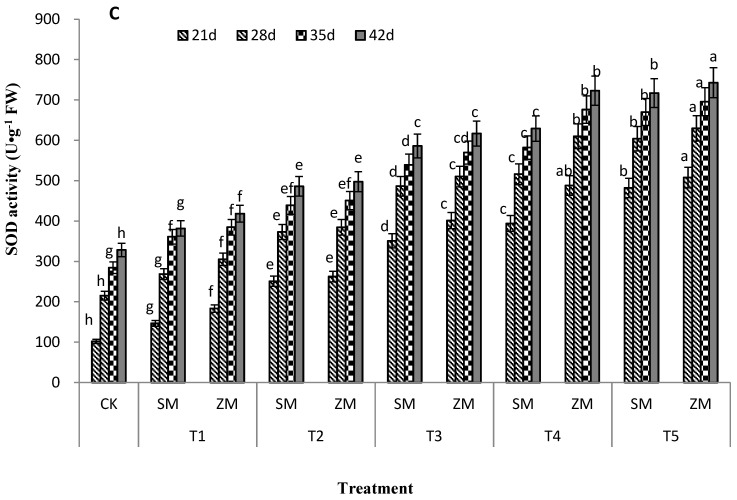
Effects of two types of wood vinegar (SM and ZM) on antioxidant enzyme activities in tomato leaves under biotic stress. Catalase (CAT) (**A**), peroxidase (POD) (**B**), and superoxide dismutase (SOD) (**C**) activities in tomato leaves. SM: Wood vinegar prepared from a single raw material; ZM: Wood vinegar prepared from different raw materials. Data are the means of six replicates (means ± standard errors). Different lowercase letters indicate significant differences between the treatments at *p* < 0.05.

Crops respond to different abiotic (pesticides, heavy metals, drought, and saline–alkali soils) and biotic (plant pathogens) stresses in the environment by triggering antioxidant defense systems [44,45,46]. This defense system is mainly composed of POD, SOD, CAT, and other proteins, which work together to improve the ability of plants to resist environmental stress. The antioxidant defense system plays a crucial role in stress-induced ROS clearance and detoxification [47,48]. As the first line of defense against ROS, SOD enhances plant resistance to environmental stress [49]. SOD effectively scavenges for O^2−^ free radicals in plants and converts the free radicals to H_2_O_2_ and molecular oxygen. H_2_O_2_ is further reduced to nontoxic H_2_O and O_2_ by CAT and POD [47]. The main role of CAT is to remove H_2_O_2_ catalyzed by SOD, glycolic acid oxidase, and uric acid oxidase. POD assists CAT in removing excess H_2_O_2_ and other peroxides [50], and counteracts the effects of stress by strengthening the cell wall, mediating signal transduction, and converting H_2_O_2_ accumulated under oxidative stress into H_2_O to remove harmful ROS [51,52]. In the present study, ZM and SM contained phenolic compounds, such as catechol and guaiacol, which alleviated the biotic stress induced by TFW and increased CAT, POD, and SOD activities (Figure 4). This finding is consistent with those of previous studies [53], POD, SOD, and CAT activities in plant seedlings treated with wood vinegar have been shown to increase under stress, which significantly improves crop growth. Phenolic compounds in wood vinegar enhance antioxidant activity by scavenging ROS free radicals, reducing power, and antilipid peroxidation [54,55]. The results of this study showed that the catechol content of ZM was significantly higher than that of SM; therefore, ZM induced higher rates of antioxidant enzyme activity in tomato leaves. Zhou showed that the effect of different wood vinegars on improving enzyme activity under stress varied considerably, which could be attributed to the variation in the contents of their chemical components and the corresponding active ingredients. The results of this study indicated that appropriate concentrations of wood vinegar prepared from different raw materials can reduce internal oxidative stress damage under biotic stress, thereby providing a favorable environment for plant growth and development. Wood vinegar prepared from different raw materials has the potential to improve the growth performance of tomato plants under biotic stress and can be generalized to other major crops. In addition, this study provided a basis for exploring the effects of wood vinegar solutions prepared from combinations of different raw materials on other biotic stressors that limit crop development and productivity.

## 4. Materials and Methods

### 4.1. Materials

#### 4.1.1. Wood Vinegar Material

The two types of wood vinegar used in this study were obtained from Kunyu Environmental Development Co., Ltd. (Kunming, China). One type of wood vinegar was prepared by mixed pyrolysis of grape, Chinese fir, and corn straw at a mass ratio of 1:1:1 (ZM), and the other type was prepared by the pyrolysis of a single raw material, Chinese fir (SM). The chemical constituents of the ZM and SM wood vinegars were analyzed using GC-MS. The relative contents of the main chemical components are listed in Tables S1 and S2.

#### 4.1.2. Plant Material

Seeds of tomato (*S. lycopersicum* L.) cultivar Bonny Best (*F. oxysporum susceptible varieties*) were purchased from Shouguang Kaijiate Agricultural Technology (Jinan, China). Seeds were disinfected with 10% sodium hypochlorite for 10 min, thoroughly rinsed three times with sterile distilled water, sterilized with 70% ethanol for 5 min, and finally rinsed five times with sterile distilled water. After germination, the seeds were sown in seedling pots and grown for pot experiments after three weeks.

#### 4.1.3. Experimental Soil

The soil used for the experiments was a mixture of soil from the back mountain of Yunnan Agricultural University in Kunming, China and fermented sheep manure at a mass ratio of 7:3.

#### 4.1.4. Pathogen Strain Tested

The pathogen strain used in this study was *F. oxysporum f*. sp. *radicis lycopersica* (FORL) and was supplied by the Laboratory of Soil Microbiology, College of Resources and Environment, Yunnan Agricultural University, Kunming, China. Pathogen spore suspension was prepared by activating the TFW pathogen on potato dextrose agar (PDA) medium (potato 200 g, glucose 20 g, agar 10 g, and distilled water 1000 mL; pH 5.98) at 28 °C for three days. Afterward, the suspension was transferred to a PDA medium, cultured at 28 °C for seven days, and filtered. The number of spores in the suspension was recorded using blood counting chamber and the spore suspension was diluted to 1 × 10^5^ spores·mL^−1^ for subsequent use.

### 4.2. Experimental Design

Experiments were conducted in a greenhouse at the College of Resources and Environment of Yunnan Agricultural University, Kunming, China, in March 2023 (spring). The temperature in the greenhouse was (25 ± 3) °C and (20 ± 2) °C during the day and at night, respectively. The light cycle was 13 h. Tomato seeds with consistent germination were planted in disposable black plastic nutrition bowls (25 cm in diameter at the bottom of the pot, 25 cm in height, and 7.5 kg of soil per pot). Two to three tomato seedlings were transplanted in each pot and then fixed after two weeks of growth. One healthy seedling with the same growth vigor was retained in each pot for the root irrigation treatment. Each tomato seedling was inoculated with 20 mL of diluted *F. oxysporum* spore suspension. After 24 h of incubation, different concentrations of SM and ZM wood vinegar were applied to the seedling roots. Each tomato seedling was irrigated with 20 mL of the root extract. Six concentration gradients were established for both types of wood vinegar: 0% (CK), 0.3% (T1), 0.6% (T2), 0.9% (T3), 1.2% (T4), and 1.5% (T5). Each treatment had six replicates for a total of 72 treatments. Distilled water was applied daily during the growth period and the soil field capacity was maintained at approximately 80% using the weighing method.

### 4.3. Indicators and Methods of Determination

The status of TFW was assessed on the 21st, 28th, 35th, and 42nd day after inoculation of tomato plants with FORL. The degree of disease was determined, and plant height, stem diameter, and leaf area were measured. Plant height was defined as the height between the base and the plant growth point, and was measured with a meter ruler; stem diameter was measured at 1 cm above the cotyledon using a digital Vernier caliper; leaf area was measured using the second leaf above the cotyledon; and leaf length (L) was measured from the leaf base (at the junction with the main stem) to the leaf tip. Leaf width (D) was determined by measuring the width of the leaf using a ruler and leaf area was calculated using formula S = L × D × 0.5468. Fresh tomato leaves at four growth stages were collected from each treatment and stored in a refrigerator at −4 °C. To allow for tomato plants to reach fruit maturity, the yield of the first three panicles of each tomato plant was counted. Six representative mature fruits from the second ear were selected based on their size and color. After surface disinfection with 70% ethanol, the fruits were stored in a refrigerator at −20 °C for further analysis.

#### 4.3.1. Determination of Disease Severity Index

The disease classification standards were as follows [56]: Grade 0: asymptomatic; Grade 1: 1 or 2 leaves turned yellow and fell off; Grade 2: 3 or 4 true leaves turned yellow, leaves wilted, and drooped; Grade 3: 5 or 6 true leaves turned yellow or true leaves wilted and drooped; and Grade 4: the whole plant wilted severely until death. The formula used was as follows [10,29]:

Disease Index (DI) = ∑(number of diseased leaves at all levels × representative grade value)/(total number of leaves surveyed × highest representative grade value) × 100.

Control Effect = (1 − disease index of test area/disease index of control area) × 100%.

#### 4.3.2. Determination of Fruit Nutritional Quality

All frozen tomato fruits were thawed at room temperature and their soluble sugar content was determined using anthrone colorimetry [57]. Tomato fruit samples (0.5 g) were ground to a homogenate, 10 mL of 80% ethanol was added, incubated in a water bath at 80 °C for 40 min, and centrifuged at 4000× *g* r·min^−1^ for 10 min. The supernatant was mixed with activated carbon in a water bath for 30 min, filtered. Afterward, 0.2 mL of the sample solution was mixed with 0.8 mL distilled water and 5 mL anthrone reagent, boiled in a water bath for 10 min, and absorbance was measured at 625 nm using a spectrophotometer. Sugar content was determined from a standard curve (the composition of the standard curve: anthrone, glucose, and distilled water) and expressed as mg.g^−1^. Soluble protein content was determined using the Coomassie brilliant blue dye binding method [58]. Fruit samples (2 g) were weighed and ground into a homogenate in distilled water (8 mL), centrifuged at 4000× *g* r·min^−1^ for 1 min, and 1 mL of the sample solution was mixed with 5 mL of Coomassie brilliant blue G-250 solution and allowed to stand for 5–20 min. Absorbance of the solution was measured at 595 nm and the protein content was determined using a standard curve (the composition of the standard curve: bovine serum albumin, Coomassie brilliant blue, and distilled water), and expressed as mg·g^−1^. Vitamin C content was quantitatively determined using 2,6-dichlorophenol indophenol titration [59]. Fruit samples (10 g) were weighed and ground in 5 mL 2% oxalic acid solution. The solution was filtered and 10 mL of the filtrate was titrated with a standard 2,6-dichlorophenol indophenol solution until a pink color persisted. Vitamin C content was calculated based on the titrated volume and expressed as mg·g^−1^. Organic acid content was determined by acid–base titration. Fruit samples (5 g) were weighed, ground, homogenized in distilled water (8 mL), incubated in a water bath at 80 °C for 30 min, and the volume was made up to 50 mL. Subsequently, 3–5 drops of phenolphthalein indicator were added to the 10 mL sample solution and titrated with 0.1 mol·L^−1^ standard sodium hydroxide solution until a reddish color persisted. Organic acid content was calculated based on the titrated volume and expressed as mg·g^−1^.

#### 4.3.3. Determination of MDA and H_2_O_2_ Contents in Tomato Leaves

MDA is a key product of membrane lipid peroxidation and its concentration reflects the extent of lipid peroxidation. MDA content was determined according to the method described by Hodges et al. [60]. Briefly, ground seedling samples (0.1 g) were homogenized in 1 mL of 0.1% (*w*/*v*) trichloroacetic acid (TCA). The homogenate was centrifuged at 16,000× *g* and 4 °C for 10 min. A 500 μL aliquot of the supernatant was added to an equal amount of 0.5% thiobarbituric acid in 20% TCA. The mixture was incubated at 95 °C for 30 min, cooled on ice, and centrifuged at 12,000× *g* for 5 min. The absorbance of the supernatant was measured at 532 nm and nonspecific absorption was measured at 600 nm. MDA concentration was calculated from the extinction coefficient 155 m/M cm using equation C = [Abs (535 − 600) ÷ 155,000] × 10^6^. MDA concentration was expressed as nmol MDA g^−1^ fresh weight (FW). H_2_O_2_ content was determined by spectrophotometry after reaction with potassium iodide (KI), according to the method described by Alexieva et al. [61]. Briefly, 0.1 g of tomato leaf samples were homogenized in 1 mL of 0.1% (*w*/*v*) TCA and the mixture was centrifuged at 16,000× *g* and 4 °C for 10 min. The supernatant, 200 μL, was mixed with 200 μL 100 mM potassium phosphate buffer (pH 7.0) and 800 μL 1 M KI (1 m KI (*w*/*v*) in fresh double distilled water H_2_ O). The reaction mixture was incubated in the dark for 1 h. Absorbance was measured at 390 nm in the absence of leaf extracts, with 0.1% TCA as blank control. H_2_O_2_ levels were determined using a predetermined H_2_O_2_ standard curve and expressed as (m mol·g^−1^ FW).

#### 4.3.4. Determination of Antioxidant Enzyme Activity in Leaves

SOD, POD, and CAT are crucial antioxidant enzymes that scavenge ROS produced in plants and prevent the potential damage caused by ROS to plant cell membrane structures. Determination of CAT activity was carried our in the following way: Frozen tomato leaves (0.5 g) were ground in a mortar containing a phosphate buffer solution (pH 7.0). The samples were centrifuged at 17,500× *g* and 4 °C for 15 min. The supernatant (100 µL) was mixed with 1.9 mL phosphate buffer (pH 7.0) and 0.01% H_2_O_2_ (1 mL). The enzyme activity was measured at 240 nm using a UV/Vis spectrophotometer (Implen P300, Duren, Germany) [62]. CAT activity was expressed as (U·mg^−1^ protein·min^−1^). Determination of POD activity was carried our as follows: Frozen tomato leaves (0.5 g) were ground in a mortar containing phosphate buffer solution (pH 7.0), and the samples were centrifuged at 17,500× *g* and 4 °C for 15 min. The supernatant (100 µL) was mixed with 2.7 mL phosphate buffer (pH 7.0), followed by the addition of 0.04% MnCl_2_ (20 µL) and 0.14% NADH (150 µL) solutions. The enzyme activity was measured at 340 nm using a UV/Vis spectrophotometer (Implen P300, Duren, Germany) [62]. POD activity was expressed as (U·mg^−1^ protein·min^−1^). Determination of SOD activity was performed in the following way: Frozen tomato leaves (0.5 g) were ground in a mortar containing a phosphate buffer solution (pH 7.8). The samples were centrifuged at 17,500× *g* and 4 °C for 15 min. The supernatant (50 µL) was mixed with 2.2 mL phosphate buffer (pH 7.8) and 0.006% riboflavin (250 µL), followed by the addition of 0.0023% methionine (250 µL) and 0.06% NBT (250 µL) solutions. The enzyme activity was measured at 560 nm using a UV/Vis spectrophotometer (Implen P300, Duren, Germany) [62]. SOD activity was expressed as (U·mg^−1^ protein).

### 4.4. Statistical Analysis

IBM SPSS Statistics 20.0 (IBM Corp., Armonk, NY, USA) was used for data analysis. The data were plotted in charts, and the means and standard deviations were calculated using Microsoft Excel 2010 software (Microsoft Corp., Redmond, WA, USA). The effects of different concentrations of the two types of wood vinegar on TFW, agronomic traits of tomato plants, membrane lipid peroxidation, H_2_O_2_ content, and antioxidant enzyme activity of tomato leaves were evaluated using a single-factor analysis of variance. Duncan’s test was used to evaluate significant differences between treatments (*p* < 0.05). Pearson’s correlation analysis was performed on the tomato disease index and agronomic traits (plant height, stem diameter, and leaf area), MDA content, H_2_O_2_ content, and leaf antioxidant enzyme activity. Pearson’s correlation analysis was also performed to determine correlations between ZM and SM concentrations and tomato disease index, agronomic traits (plant height, stem diameter, and leaf area), MDA content, H_2_O_2_ content, and leaf antioxidant enzyme activity.

## 5. Conclusions

Different concentrations of ZM and SM significantly affected tomato plants infected with TFW. The two types of wood vinegar promoted tomato growth and development, reduced membrane lipid peroxidation, reduced H_2_O_2_ content of leaves, and induced antioxidant enzyme activity in tomato leaves under biotic stress. The response intensity of the two types of wood vinegar to growth, development, physiology, and biochemistry of tomatoes was in the order of ZM > SM. In addition, a concentration of 1.5% wood vinegar induced stress in tomato plants. The contents of the main phenolic components in ZM wood vinegar were significantly higher than those in SM wood vinegar. Therefore, ZM wood vinegar can enhance the disease resistance of plants by promoting plant growth and development, reducing the degree of membrane lipid peroxidation in plant leaves, reducing H_2_O_2_ content, and inducing antioxidant enzyme activity in plant leaves. The results of this study provide not only insights into the high value utilization of biomass resources, but also a theoretical basis for improving crop production.

## Figures and Tables

**Table 1:** Effects of two types of wood vinegar (SM and ZM) on tomato seedling growth and development.

Treatment		Plant Height (cm)				Stem Diameter (mm)				Leaf Area (cm^2^)			
		21 d	28 d	35 d	42 d	21 d	28 d	35 d	42 d	21 d	28 d	35 d	42 d
Control	CK	4.63 ^f^	5.69 ^g^	7.63 ^f^	10.03 ^f^	2.62 ^d^	3.72 ^f^	3.97 ^d^	4.96 ^g^	1.00 ^e^	1.11 ^g^	1.27 ^f^	1.57 ^f^
T1	SM	5.59 ^de^	7.09 ^ef^	9.03 ^e^	10.93 ^e^	2.63 ^d^	3.73 ^f^	3.98 ^d^	4.94 ^g^	1.24 ^de^	1.37 ^e^	1.52 ^e^	1.82 ^e^
	ZM	6.06 ^c^	7.56 ^de^	9.50 ^de^	11.37 ^de^	2.70 ^cd^	3.86 ^e^	4.04 ^cd^	5.12 ^e^	1.43 ^cd^	1.56 ^d^	1.71 ^d^	2.01 ^d^
T2	SM	5.84 ^cd^	7.77 ^d^	9.80 ^d^	11.70 ^d^	2.95 ^bc^	4.05 ^d^	4.30 ^b^	5.29 ^d^	1.13 ^de^	1.26 ^f^	1.42 ^e^	1.71 ^e^
	ZM	6.89 ^b^	8.53 ^c^	10.47 ^c^	12.43 ^c^	2.97 ^b^	4.13 ^c^	4.32 ^b^	5.47 ^c^	1.83 ^b^	1.96 ^b^	2.10 ^b^	2.41 ^b^
T3	SM	7.47 ^a^	9.27 ^b^	11.27 ^b^	13.40 ^b^	3.26 ^ab^	4.56 ^b^	4.81 ^a^	5.80 ^b^	1.66 ^bc^	1.79 ^c^	1.93 ^c^	2.24 ^c^
	ZM	7.71 ^a^	10.24 ^a^	12.37 ^a^	14.37 ^a^	3.54 ^a^	4.77 ^a^	4.89 ^a^	5.91 ^a^	2.22 ^a^	2.28 ^a^	2.43 ^a^	2.73 ^a^
T4	SM	5.46 ^e^	6.96 ^f^	8.90 ^e^	10.80 ^e^	2.67 ^cd^	3.77 ^f^	4.02 ^cd^	5.01 ^fg^	1.07 ^de^	2.03 ^b^	2.18 ^b^	2.41 ^b^
	ZM	5.86 ^cd^	7.36 ^def^	9.30 ^de^	11.20 ^de^	2.75 ^c^	3.85 ^e^	4.10 ^c^	5.09 ^ef^	1.91 ^ab^	2.04 ^b^	2.19 ^b^	2.49 ^b^
T5	SM	4.36 ^f^	5.46 ^g^	7.40 ^f^	9.30 ^g^	1.83 ^f^	2.93 ^h^	3.18 ^f^	4.17 ^i^	0.89 ^e^	1.05 ^g^	1.17 ^f^	1.47 ^f^
	ZM	3.46 ^g^	4.36 ^h^	6.03 ^g^	8.03 ^h^	2.14 ^e^	3.24 ^g^	3.46 ^e^	4.45 ^h^	1.08 ^de^	1.28 ^ef^	1.42 ^e^	1.72 ^e^

SM: Wood vinegar prepared from a single raw material; ZM: Wood vinegar prepared from different raw materials. Data are the means of six replicates (means ± standard errors). Different lowercase letters indicate significant differences between the treatments at *p* < 0.05.

**Table 2:** Effects of two types of wood vinegar (SM and ZM) on nutritional quality and yield of tomato fruits.

Treatment		Soluble Sugar (%)	Soluble Protein (mg·g^−1^)	Vitamin C (mg·100 g^−1^)	Organic Acid (%)	Yield (g)
Control	CK	7.87 ± 0.04 i	1.24 ± 0.11 h	15.26 ± 0.62 i	4.73 ± 0.11 b	248.65 ± 29.75 f
T1	SM	8.03 ± 0.02 h	1.42 ± 0.06 g	17.16 ± 0.45 h	2.21 ± 0.06 g	342.23 ± 33.29 bcde
	ZM	8.85 ± 0.02 e	2.13 ± 0.11 f	22.84 ± 0.70 f	5.22 ± 0.14 a	363.34 ± 45.09 bcd
T2	SM	8.17 ± 0.03 g	2.11 ± 0.11 f	18.92 ± 0.22 g	3.12 ± 0.11 e	358.60 ± 36.06 bcd
	ZM	9.04 ± 0.04 d	3.48 ± 0.10 d	30.20 ± 0.63 d	3.51 ± 0.06 d	391.09 ± 25.70 bc
T3	SM	8.75 ± 0.04 f	3.30 ± 0.06 e	22.97 ± 0.22 f	2.58 ± 0.06 f	432.23 ± 35.25 b
	ZM	9.58 ± 0.01 c	4.28 ± 0.08 b	33.09 ± 0.32 c	3.76 ± 0.09 d	553.55 ± 84.66 a
T4	SM	8.82 ± 0.04 e	3.45 ± 0.06 d	25.02 ± 0.72 e	3.41 ± 0.57 de	316.61 ± 51.75 cdef
	ZM	9.81 ± 0.04 b	4.85 ± 0.10 a	35.49 ± 1.15 b	3.76 ± 0.14 d	251.63 ± 40.84 ef
T5	SM	8.99 ± 0.04 d	3.63 ± 0.08 c	25.89 ± 0.44 e	4.39 ± 0.01 c	376.19 ± 44.06 bcd
	ZM	10.04 ± 0.04 a	4.96 ± 0.06 a	39.54 ± 0.95 a	3.56 ± 0.06 d	288.57 ± 80.34 def

SM: Wood vinegar prepared from a single raw material; ZM: Wood vinegar prepared from different raw materials. Data are the means of six replicates (means ± standard errors). Different lowercase letters indicate significant differences between the treatments at *p* < 0.05.

**Table 3:** Pearson correlation analysis (R) of tomato disease severity with plant agronomic traits (plant height, stem diameter and leaf area), MDA content, H_2_O_2_ content and disease resistance-related enzyme activities.

	Disease Index	Plant Height	Stem Diameter	Leaf Area	MDA	H_2_O_2_	CAT	POD
Plant Height	−0.69 **							
Stem Diameter	−0.59 **	0.81 **						
Leaf Area	−0.71 **	0.85 **	0.93 **					
MDA	0.82 **	−0.67 **	−0.73 **	−0.69 **				
H_2_O_2_	0.51 **	−0.52 **	−0.47 **	−0.39 **	0.79 **			
CAT	−0.40 *	0.34	0.19	0.21	−0.63 **	−0.72 **		
POD	0.01	−0.15	−0.20	−0.31	−0.29	−0.63 **	0.52 **	
SOD	−0.02	−0.20	−0.20	−0.33	−0.35 *	−0.62 **	0.48 **	0.94 **

** Significant difference (*p* < 0.01); * significant difference (*p* < 0.05).

**Table 4:** Pearson correlation analysis (R) of ZM concentration with tomato disease severity, plant agronomic traits (plant height, stem thickness, and leaf area), MDA content, H_2_O_2_ content, and disease resistance-related enzyme activities.

	Concentration of ZM	Disease Index	Plant Height	Stem Diameter	Leaf Area	MDA	H_2_O_2_	CAT	POD
Disease Index	0.73 **								
Plant Height	−0.54 *	−0.85 **							
Stem Diameter	−0.43	−0.77 **	0.97 **						
Leaf Area	−0.19	−0.57 *	0.88 **	0.89 **					
MDA	0.32	0.57 *	−0.88 **	−0.88 **	−0.96 **				
H_2_O_2_	−0.48	0.14	−0.41	−0.53 *	−0.62 *	0.43			
CAT	0.83 **	0.27	0.01	0.13	0.31	−0.14	−0.86 **		
POD	0.96 **	0.63 *	−0.46	−0.35	−0.15	0.32	−0.56 *	0.83 **	
SOD	0.98 **	0.69 **	−0.42	−0.31	−0.04	0.15	−0.53 *	0.86 **	0.91 **

** Significant difference (*p* < 0.01); * significant difference (*p* < 0.05).

**Table 5:** Pearson correlation analysis (R) of SM concentration with tomato disease severity, plant agronomic traits (plant height, stem thickness, and leaf area), MDA content, H_2_O_2_ content, and disease resistance-related enzyme activities.

	Concentration of SM	Disease Index	Plant Height	Stem Diameter	Leaf Area	MDA	H_2_O_2_	CAT	POD
Disease Index	0.04								
Plant Height	−0.45	−0.84 **							
Stem Diameter	−0.50	−0.85 **	0.97 **						
Leaf Area	−0.46	−0.59 *	0.86 **	0.81 **					
MDA	−0.04	0.89 **	−0.85 **	−0.79 **	−0.76 **				
H_2_O_2_	−0.34	0.81 **	−0.62 *	−0.57 *	−0.46	0.88 **			
CAT	0.29	−0.85 **	0.74 **	0.70 **	0.64 *	−0.84 **	−0.71 **		
POD	0.89 **	−0.23	−0.18	−0.24	−0.26	−0.23	−0.39	0.42	
SOD	0.98 **	−0.07	−0.33	−0.40	−0.36	−0.14	−0.41	0.21	0.94 **

** Significant difference (*p* < 0.01); * significant difference (*p* < 0.05).

## Data Availability

Data are contained within the article.

## Supplementary Materials

The following supporting information can be downloaded at: https://www.mdpi.com/article/10.3390/plants13020157/s1, Table S1: Relative content of main compounds in ZM wood vinegar; Table S2: Relative content of main compounds in SM wood vinegar.
