# Supplementary material for: Physiological and Biochemical Mechanisms of Wood Vinegar-Induced Stress Response against Tomato Fusarium Wilt Disease
Source: Plants (Basel). 2024 Jan 6;13(2):157. doi: 10.3390/plants13020157 (PMC10819440; doi:10.3390/plants13020157)
Supplement: Supplementary file 1 [file plants-13-00157-s001.zip › plants-2783366-supplementary.pdf]

Table S1: Relative content of main compounds in ZM wood vinegar

Group	Compound name	Chemical formula	R.T. /min	Area (%)
**Acids**	N-Ethylglycine	$C_4H_9NO_2$	12.42	18.46
	lactic acid	$C_3H_6O_3$	7.42	14.3
	3-Hydroxypyridine	C_5H_5NO	8.61	10.25
	palmitic acid	$C_{16}H_{32}O_2$	19.32	4.9
	2-ketoadipate	$C_6H_8O_5$	10.05	3.46
	glycolic acid	$C_2H_4O_3$	7.68	2.12
	stearic acid	$C_{18}H_{36}O_2$	21.11	1.64
	adipic acid	$C_6H_{10}O_4$	13.38	1.37
	Minors	——	——	0.9
**Phenols**	catechol	$C_6H_6O_2$	11.06	13.14
	guaiacol	$C_7H_8O_2$	9.84	11.04
	m-cresol	C_7H_8O	8.63	1.64
	o-cresol	C_7H_8O	8.50	0.34
	Minors	——	——	0.29
**Ketones**	1,3-Cyclohexanedione	$C_6H_8O_2$	10.74	1.03
	1,2-Cyclohexanedione	$C_6H_8O_2$	15.33	1.03
	Minors	——	——	0.41
**Esters**	Methyl jasmonate	$C_{13}H_{20}O_3$	16.17	1.19
	D-erythronolactone	$C_4H_6O_4$	12.47	0.1
	Minors	——	——	0.04
**Aldehydes**	succinate semialdehyde	$C_4H_6O_3$	9.20	3.25
	glutaraldehyde 3	$C_5H_8O_2$	10.54	0.2
	2,5-dihydroxybenzaldehyde	$C_7H_6O_3$	15.94	0.17
	butyraldehyde	C_4H_8O	10.50	0.15
	Minors	——	——	0.03
**Alcohols**	Perillyl Alcohol	$C_{10}H_{16}O$	12.21	1.96
	3-Methylamino-1,2-propanediol	$C_4H_{11}NO_2$	9.46	0.44
	(+)-Dihydrocarveol	$C_{10}H_{18}O$	10.72	0.29
	3-Methylbenzyl Alcohol	$C_8H_{10}O$	10.00	0.13
	Acetol	$C_3H_6O_2$	13.71	0.12
	Minors	——	——	0.26
**Undetected substances**	——	——	——	5.66

Table S2: Relative content of main compounds in SM wood vinegar

Group	Compound name	Chemical formula	R.T. /min	Area(%)
**Acids**	palmitic acid	$C_{16}H_{32}O_2$	19.32	12.93
	lactic acid	$C_3H_6O_3$	7.42	8.73
	picolinic acid	$C_6H_5NO_2$	11.09	5.68
	2-ketoadipate	$C_6H_8O_5$	10.05	4.56
	N-Ethylglycine	$C_4H_9NO_2$	12.42	4.49
	adipic acid	$C_6H_{10}O_4$	13.38	2.99
	stearic acid	$C_{18}H_{36}O_2$	21.11	2.98
	glycolic acid	$C_2H_4O_3$	7.68	1.77
	2-hydroxybutanoic acid	$C_4H_8O_3$	8.33	0.64
	Minors	——	——	1.6
**Phenols**	guaiacol	$C_7H_8O_2$	9.84	11.39
	catechol	$C_6H_6O_2$	11.06	9.32
	m-cresol	C_7H_8O	8.63	2.82
	o-cresol	C_7H_8O	8.50	1.29
	Minors	——	——	0.14
**Ketones**	1,3-Cyclohexanedione	$C_6H_8O_2$	10.74	3.56
	1,2-Cyclohexanedione	$C_6H_8O_2$	15.33	2.38
	Minors	——	——	0.13
**Esters**	D-erythronolactone	$C_4H_6O_4$	12.47	3.14
	Linoleic acid methyl ester	$C_{19}H_{34}O_2$	19.77	1.01
	Minors	——	——	0.09
**Aldehydes**	glutaraldehyde	$C_5H_8O_2$	10.54	1.23
	2,5-dihydroxybenzaldehyde	$C_7H_6O_3$	15.94	1.11
	succinate semialdehyde	$C_4H_6O_3$	9.20	1.1
	Minors	——	——	0.06
**Alcohols**	Perillyl Alcohol	$C_{10}H_{16}O$	12.21	3.54
	3-Methylbenzyl Alcohol	$C_8H_{10}O$	10.00	0.58
	(+)-Dihydrocarveol	$C_{10}H_{18}O$	10.72	0.44
	3-Methylamino-1,2-propanediol	$C_4H_{11}NO_2$	9.46	0.29
	Acetol	$C_3H_6O_2$	13.71	0.29
	Minors	——	——	0.16
**Undetected substances**	——	——	——	10.78
